# Colon cancer of Peutz-Jeghers syndrome with gallolyticus endocarditis

**DOI:** 10.1007/s12328-019-01080-9

**Published:** 2019-12-16

**Authors:** Kiyoshi Miyahara, Shunichi Tobe, Tatsunori Shizuku, Rin Inamoto, Ikuo Katayama

**Affiliations:** 1 Department of General Internal Medicine, Shonan Fujisawa Tokushukai Hospital, Tujido-Kandai 1-5-1, Fujisawa, Kanagawa Japan; 2 Center of Hepato-Gastroenterology, Shonan Fujisawa Tokushukai Hospital, Fujisawa, Kanagawa Japan; 3 Department of Cardiovascular Surgery, Shonan Fujisawa Tokushukai Hospital, Fujisawa, Kanagawa Japan

**Keywords:** Peutz-Jeghers syndrome, Colon cancer, *Streptococcus gallolyticus*, Infective endocarditis

## Abstract

We report a case of Peutz-Jeghers syndrome with gallolyticus endocarditis which has not yet been reported. Colon cancer was observed and implicated in Peutz-Jeghers syndrome. A 44-year-old female with fever and heart murmur was diagnosed as infective endocarditis caused by streptococcus gallolyticus. After treatment with antibiotics and mitral valbuloplasty, we performed gastrointestinal endoscopic studies and found polyps in stomach and colon. Histological findings of a large pedunculated colon polyp revealed hamartomatous polyp with a lesion of adenocarcinoma with adenoma. She had pigmentation of digits. Her father had also digits pigmentation and died of pancreas cancer. Peutz-Jeghers syndrome with colon cancer was incidentally diagnosed by infective endocarditis and subsequent colonoscopy.

## Introduction

Peutz-Jeghers syndrome (PJS) is an autosomal dominant inherited disorder with germline mutation in serine/threonine kinase-11 (STK11) gene and characterized by intestinal hamartomatous polyps with skin melanocytes macules. The estimated incidence of the disease is approximately 1 in 50,000–200,000 births [1]. The clinical symptoms are non-specific such as anemia, nausea, abdominal pain and intestinal intussusception. Accordingly, it is hard to come to a diagnosis without gastrointestinal endoscopic study. Patients with PJS have an increased risk of cancer in colon, pancreas, lung, breast and ovary [2, 3].

Infective endocarditis is a potentially lethal disease and the incidence is from 1.5 to 11.6 cases per 100,000 people [4]. 7% of all kinds of infective endocarditis are caused by *Streptococcus gallolyticus* [5]. Gallolyticus endocarditis is well recognized to be accompanied by colon cancer and endoscopic study of colon has been recommended [6].

We recently encountered an adult case of Peutz-Jeghers type hamartoma with colon cancer associated with gallolyticus endocarditis.

## Case report

**Fig. 1:**
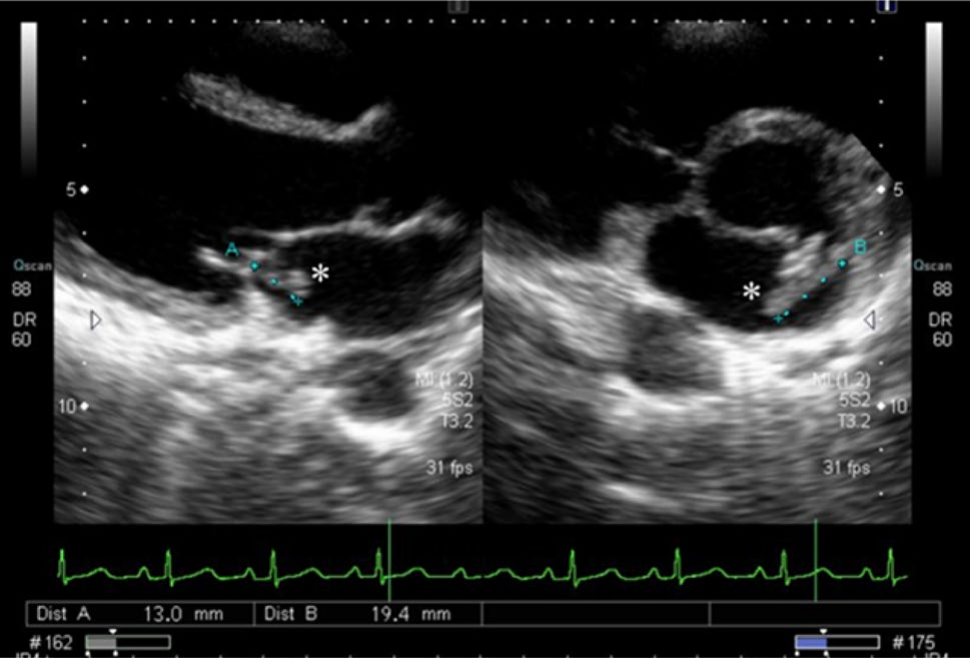
Transthoracic echocardiogram. Left image is long-axis tomogram and right image is short-axis tomogram. There is a large soft vegetation (astreik) on mitral valve

**Fig. 2:**
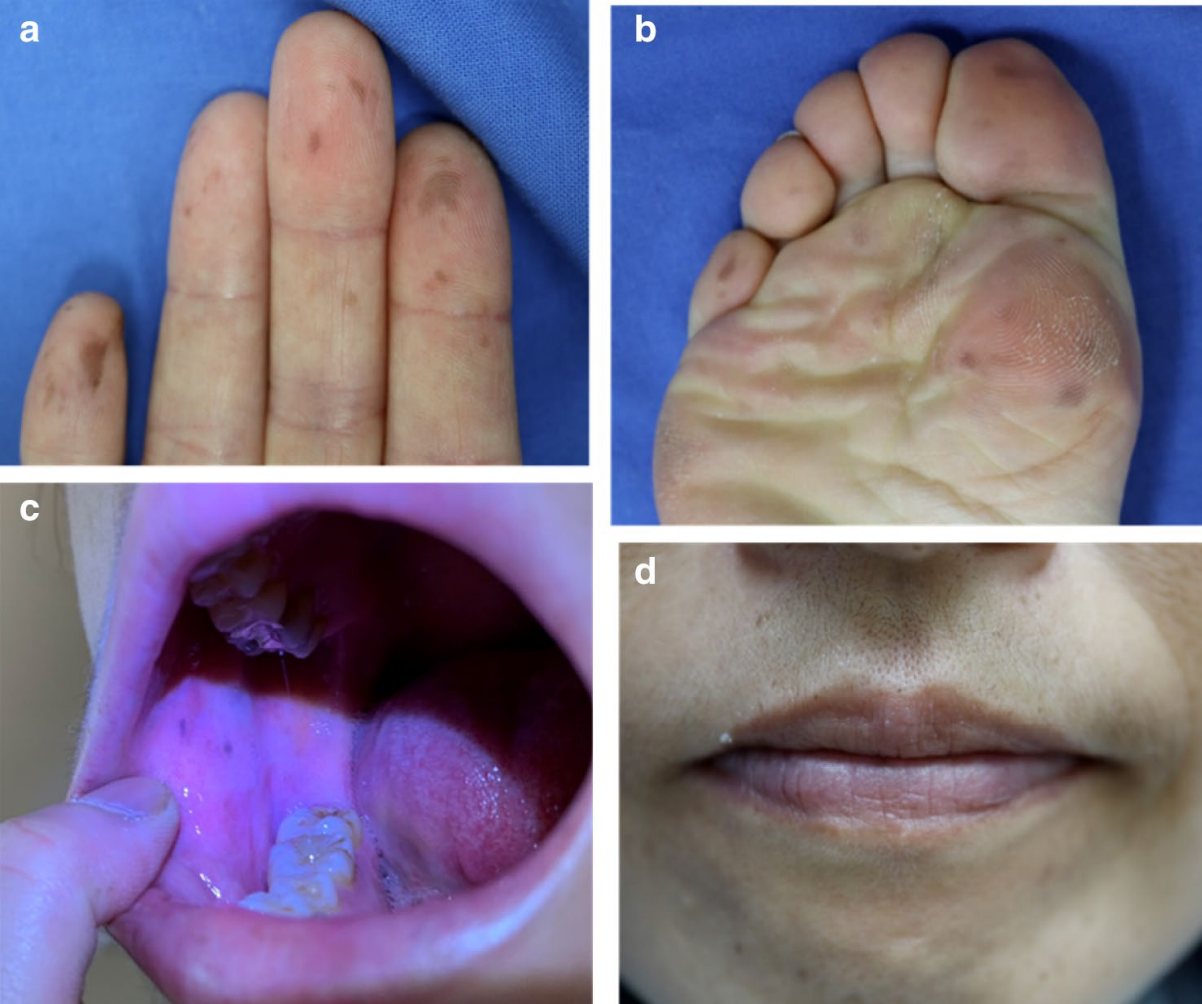
Skin melanocytes macules of hands and feet, and pigmentation of buccal mucosa. **a** Disseminated pigmentation in various size is visible on the right fingers. **b** Skin pigmentation on the toes and sole of the left foot. **c** Melanotic pigmentation of right buccal mucosa is visible. **d** No pigmentation on lips

A 44-year-old female with a recent onset of night chills and fever was referred to our hospital and diagnosed as infective endocarditis because of mitral regurgitation on auscultation and mitral valve vegetation on transthoracic echocardiography (Fig. 1). Laboratory values included total protein level of 7.0 g/dl, C-reactive protein level of 5.39 mg/dl, erythrocyte sedimentation rate of 45 mm/h, white-cell count of 4700/µl with 85.2% neutrophils, hemoglobin level of 9.6 g/dl, mean corpuscular volume of 73.6 fl, reticulocyte count of 30,900/µl, iron level of 12 µg/dl, ferritin level of 55.0 ng/ml and platelet count of 161,000/µl. She was admitted to our hospital and received intravenous medication of ceftriaxone and vancomycin. *S. gallolyticus* was identified in blood culture. Physical examination revealed neither Janeway lesions nor Osler nodes. Skin pigmentation of bilateral fingers and soles had existed without pain since childhood. Pigmentation of buccal mucosa was visible but that of lips was not (Fig. 2).

Two days later, after transfusion of eight units of red cell concentrate, mitral valvuloplasty was performed successfully by cardiovascular surgeons. Microscopic findings of resected mitral valve showed bacterial colonization on the surface of thrombus. Three weeks later, ceftriaxone was discontinued due to adverse effects and ampicillin had been administered intravenously for six consecutive weeks.

**Fig. 3:**
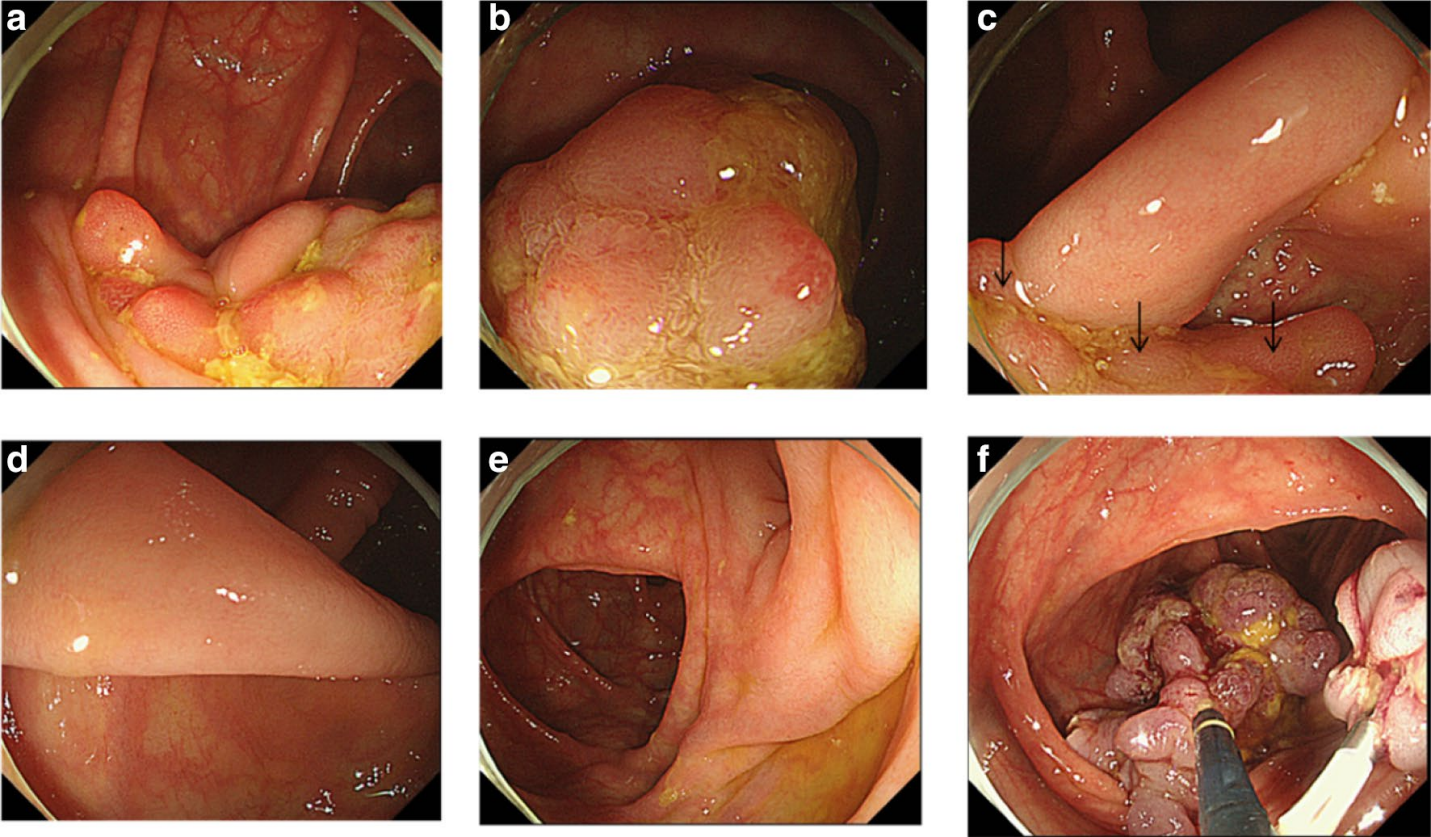
Colonoscopic findings of the large pedunculated colon polyp in the splenic flexure of colon. **a**, **b** Views of a part of polyp head. **c** The polyp neck and arrows show the part of polyp head. **d** The long stalk of pedunculated polyp. **e** The root of pedunculated polyp. **f** A view of the polyp head on the process of retrieving just after polypectomy. Small parts of the head were torn off during operation and stayed on the right side of the view

**Fig. 4:**
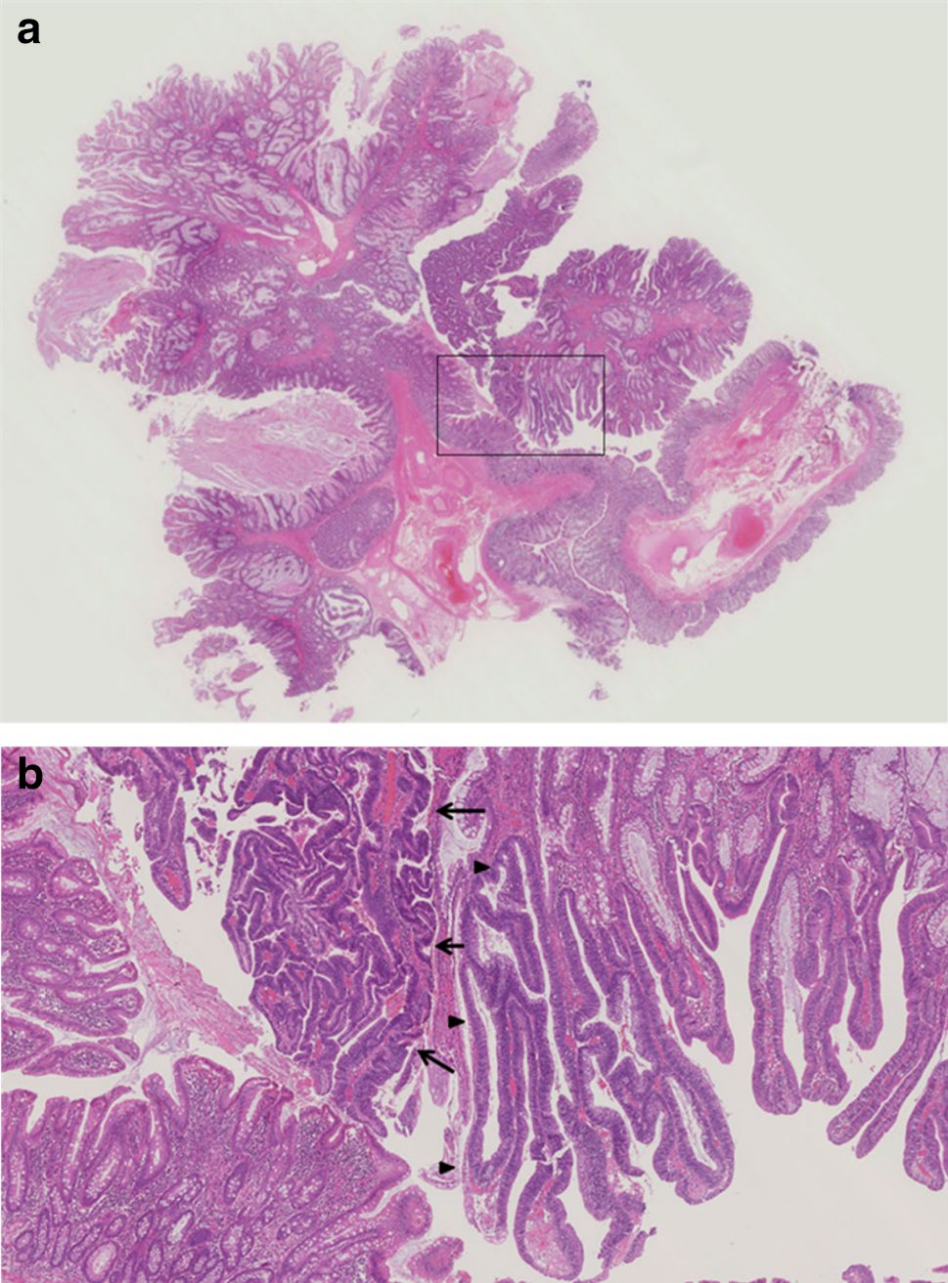
Histopathological findings of the colon polyp. **a** Low power magnification of the hamartomatous polyp of colon. **b** Enlarged boxed section of figure **a**. Hamartomatous polyp with a lesion of adenocarcinoma (arrows) with villous adenoma (arrowheads) (H and E stain)

Colonoscopy was performed on 36th hospital day because it had been reported that colon cancer is frequently associated with gallolyticus endocarditis, and revealed a pedunculated polyp in the splenic flexure of colon (Fig. 3). The large polyp with 30 mm in length was retrieved by endoscopic polypectomy and its pathological diagnosis was hamartomatous polyp with a lesion of adenocarcinoma with adenoma (Fig. 4). Laboratory data showed total protein level of 6.7 g/dl, C-reactive protein level of 0.27 mg/dl, white-cell count of 2100/µl with 45.6% neutrophils, hemoglobin level of 10.8 g/dl, mean corpuscular volume of 84.3 fl and platelet count of 181,000/µl. Upper-gastrointestinal endoscopy also revealed multiple polyps with 2–15 mm in diameter in the body of stomach. Endoscopically, twelve polyps sized less than 6 mm in diameter appeared as translucent, smooth-surfaced sessile polyps. There were three larger subpedunculated polyps with 10 mm, 12 mm and 15 mm in diameter. Endoscopic mucosal resections were performed for these three polyps, all of which were pathologically diagnosed as fundic gland polyps. We performed balloon-assisted endoscopies by oral and anal insertion to survey whole small intestine and found no polyp in duodenum, jejunum and ileum.

Her father died of pancreas cancer at age 57. He also had pigmentation of digits without a medical history of gastrointestinal disease. Her elder brother also had the same kind of skin pigmentation but had had no health problem up to that time.

Since the patient had a colon hamartomatous polyp with the characteristic pigmentation of buccal mucosa and skin, she was diagnosed as PJS according to the diagnostic criteria of European consensus statement [3]. According to her memories, perioral pigmentation had faded away with growth. We recommended that she should receive genetic testing for mutations in the STK11 gene, but she did not want it. She was in good physical condition on discharge, with laboratory values including total protein level of 7.0 g/dl, C-reactive protein level of 0.17 mg/dl, white-cell count of 2100/µl with 48.7% neutrophils and hemoglobin level of 11.6 g/dl.

## Discussion

In the case presented here, PJS and gallolyticus endocarditis coexisted, which has not been reported before. This 44-year-old female patient had colon cancer in addition to the above syndrome and disorder.

Infective endocarditis is a relatively rare but life-threating disease with acute heart failure due to valve destruction and organ failure of brain and kidney. There are many causative bacteria inducing infective endocarditis. *S. gallolyticus* accounts for 7% of them [5]. The first case of enterococcal endocarditis associated with colon cancer was reported in 1951 [7] and gallolyticus endocarditis is nowadays well known as a high risk of colon cancer [8]. *S. gallolyticus* is one of intestinal colonized bacteria. Recently, relationships between intestinal bacteria and colorectal cancer have been indicated in Fusobacterium, Peptostreptococcus, Atopobium and Actinomyces by metagenomics and metabolic analysis [9]. Currently, it is not certain in this case whether intestinal colonized *S. gallolyticus* is an additive inducer of carcinogenesis or not.

Bacteremia due to other intestinal microbes also has a high risk of colon cancer [10]. It is speculated that these bacteria enter the bloodstream with intestinal dysbiosis and perturbed barrier function. The nature and the etiological role of *S. gallolyticus* with colon neoplasm have been extensively studied in mice. It is suggested that *S. gallolyticus* might be passengers and cancer-promoting bacteria [11]. The underlying mechanisms remain to be clarified further.

In this patient, a large colon polyp was found on endoscopic screening for colon cancer and was histopathologically diagnosed as a colon hamartomatous polyp. Hamartomatous polyposis syndromes are rare genetic syndromes, which include PJS, PTEN hamartoma tumor syndrome (Cowden Syndrome and Bannayan-Riley-Ruvalcaba Syndrome) and Juvenile polyposis syndrome. PJS can be differentiated from Cowden syndrome which has mucocutaneous lesions including trichilemmomas, acral keratoses and papillomatous lesions, and from Bannayan-Riley Ruvalcaba syndrome which has macrocephaly, lipomatosis and pigmented macules of the glans penis. Pigmented spots in Cowden and Bannayan-Riley-Ruvalcaba syndrome characteristically do not occur on the lips as seen in PJS. Juvenile polyposis syndrome is characterized by multiple gastrointestinal juvenile polyps but is not associated with mucocutaneous pigmentation.

PJS is a rare autosomal dominant disorder characterized by the hamartomatous polyps in the gastrointestinal tract and by melanin spots on lips, buccal mucosa and digits. Germline mutations of the STK11 gene, located on chromosome 19p13.3, are the cause in the majority of cases with PJS [12, 13]. STK11 is a protein kinase regulating energy metabolism and proliferation of the cell and is recognized as a key enzyme of tumor suppression [14, 15].

STK11 deficiency in the gastrointestinal stroma is an important factor for the formation of polyps [16, 17], but the trigger of its mechanisms has remained unclear. Recently, a new finding has been reported; the mutations of STK11 in T cells induce inflammation in the stroma and drive polyp formation [18]. Genetic alterations of STK11 were also found to play a causal role in lung adenocarcinoma [19]. It was suggested that an overlapping of p53 mutation might increase carcinogenesis [20]. Cancer risk in PJS is high with age. The risk for developing cancer at the age of 30, 40, 50 and 60 years is 3–5%, 17–21%, 31–47% and 55–60%, respectively. Gastrointestinal cancer is the most common cancer and the risk is 28–33% at age 60 [21, 22]. Because of increased cancer risk in gastrointestinal tract, pancreas, breast and ovary, it has been recommended that patients with PJS be periodically followed to rule out cancers [1–3].

We speculated the reason that coexistence of PJS and gallolyticus endocarditis has not been reported is due to very low prevalence of each disorder in the population [1, 4]. Consequently, an extremely rare chance of their coexistence might have incidentally occurred. Additionally, most patients with PJS had undergone preventive resections of intestinal polyps in their younger ages than this patient after diagnosis of PJS [2, 3], which might have resulted in less opportunities of intestinal microbial entry into the bloodstream and subsequent infective endocarditis. The age of diagnosis of PJS is between 10 and 30 years with the average age of 26 years in women [23]. On the contrary, the skin pigmentation, only visible sign of PJS in this patient, had not been concerned by anyone until 44 years of age and resulted in the delay of its diagnosis. As the consequence, at the middle age with higher risk of colon cancer, it was observed that PJS and gallolyticus endocarditis coexisted in this patient. Therefore, it would be likely that there have been no literatures regarding their coexistence.

In summary, most patients with PJS are found in children with its clinical features such as pigmentation of lips and a family history of PJS. Pigmentation of lips usually fades away after adolescence. Accordingly, for the diagnosis of PJS in adult case, clinical signs and symptoms which lead to examine gastrointestinal tract are important clues to find hamartomatous polyps. In this case, gallolyticus endocarditis might have been a silent sign of PJS.

## Abbreviations

PJS: Peutz-Jeghers syndrome
STK11: Serine/threonine kinase-11
